# Integrated Biochemical and Computational Evaluation of Bis-Imidazolium Derivatives 3e–3g in Cholinesterase and HIV-1 Reverse Transcriptase Assays

**DOI:** 10.3390/molecules31214006

**Published:** 2026-11-06

**Authors:** Edanur Altuntas, Aleyna Nalcaoglu Senocak, Yasemin Ünver, Halil Ibrahim Guler

**Affiliations:** 1 Department of Molecular Biology and Genetics, Faculty of Science, Karadeniz Technical University, Trabzon 61080, Turkey; 2 Department of Molecular Biology and Genetics, Graduate Institute of Natural and Applied Sciences, Karadeniz Technical University, Trabzon 61080, Turkey; 3 Department of Chemistry, Faculty of Science, Karadeniz Technical University, Trabzon 61080, Turkey

**Keywords:** bis-imidazolium derivatives, acetylcholinesterase, butyrylcholinesterase, HIV-1 reverse transcriptase, molecular dynamics

## Abstract

Bis-imidazolium derivatives (**3e, 3f, 3g**) were evaluated for their inhibitory activity against acetylcholinesterase (AChE), butyrylcholinesterase (BChE), and human immunodeficiency virus type 1 reverse transcriptase (HIV-1 RT) using colorimetric assays and molecular docking. For the HIV-1 RT–**3e** complex, a 100 ns molecular dynamics (MD) simulation and Molecular Mechanics/Generalized Born Surface Area (MM/GBSA) analysis were performed. Compound **3e** emerged as the most active derivative, showing the lowest half-maximal inhibitory concentration (IC_50_) values: 13.36 µg/mL for AChE, 4.95 µg/mL for BChE, and 50.44 µg/mL for HIV-1 RT. Nevirapine produced 95.33% RT inhibition at 20 µM. Redocking validated the binding poses with root-mean-square deviation (RMSD) values below 2.0 Å, though docking scores lacked consistent correlation with experimental activity rankings. In the MD trajectory, **3e** remained stable within the RT binding pocket, yielding an MM/GBSA energy of −51.04 ± 8.39 kcal/mol. Absorption, distribution, metabolism, excretion, and toxicity (ADMET) predictions indicated low gastrointestinal absorption, no blood–brain barrier permeation, and a high-confidence human ether-à-go-go-related gene (hERG) potassium channel blockade alert. These findings highlight **3e** for further optimization.

## 1. Introduction

Alzheimer’s disease (AD) is a progressive neurodegenerative disorder characterised by cognitive and functional decline, for which the development of effective pharmacological interventions remains a major research priority. Amyloid-directed therapy has expanded the therapeutic landscape: the TRAILBLAZER-ALZ 2 trial demonstrated that donanemab slowed clinical progression in patients with early symptomatic AD and biomarker-confirmed amyloid and tau pathology [1]. Nevertheless, the persistence of impairment and the occurrence of treatment-associated adverse events [1] sustain interest in approaches that address additional aspects of the disease. Cholinergic modulation remains relevant to this effort. AChE and BChE are established targets for investigating compounds that influence acetylcholine hydrolysis. Structural studies of inhibitor-bound human AChE have provided a basis for interpreting ligand recognition within its binding gorge [2]. In parallel, recent optimisation of a selective BChE inhibitor has linked improved inhibitory potency and brain exposure with procognitive effects in animal models [3]. Together, these findings support evaluating enzyme activity alongside the molecular and physicochemical properties that influence inhibitor development.

HIV-1 reverse transcriptase (RT) represents a distinct enzyme target with a well-developed structural framework for inhibitor discovery. Its role in converting the viral RNA genome into DNA makes interference with RT activity a central strategy in antiretroviral drug development. Non-nucleoside reverse transcriptase inhibitors (NNRTIs) act through an allosteric binding pocket, where resistance-associated amino acid substitutions can compromise inhibitor activity. The search for alternative chemical architectures therefore continues to be important. Recent research on indolylarylsulfone derivatives combined biochemical screening, cellular antiviral assays, and molecular modelling to identify active compounds and examine susceptibility to resistance-associated variants [4]. This work illustrates the value of relating molecular design to experimentally measured inhibition while distinguishing activity against an isolated enzyme from antiviral efficacy in cells. Exploring additional chemical series at the enzyme level can contribute to this process by identifying activities that warrant more detailed mechanistic and cellular investigation.

Imidazole-containing structures offer a versatile chemical framework for investigating enzyme inhibition, although biological activity depends on the complete molecular architecture. An imidazole-amide biarylether series has shown antiviral activity against wild-type HIV-1 and the NNRTI-resistant Y188L variant, providing a scaffold-specific precedent for imidazole chemistry in RT-directed inhibitor design [5]. Recent studies have also demonstrated AChE inhibition by imidazolium salts carrying two positive charges [6] and by imidazolium derivatives incorporating a 2-oxindole scaffold [7]. These observations provide an experimental rationale for examining further imidazolium-containing compounds with systematic structural variation. In bis-imidazolium series, the combination of two cationic centres, an aromatic linker, and alkyl substituents permits investigation of how a shared framework accommodates distinct enzyme environments. Varying alkyl-chain length within such a series provides a focused structural comparison without changing the central scaffold.

The compounds investigated here originate from a previous study, which reported the synthesis and spectroscopic characterisation of bis-imidazolium bromides **3a**–**3g** and evaluated their antitumour and antimicrobial activities, together with docking analyses involving epidermal growth factor receptor and vascular endothelial growth factor receptor 1 [8]. Within that series, compounds **3e**, **3f**, and **3g** carry octyl, nonyl, and decyl substituents, respectively, on the same bis-imidazolium framework. Their prior biological evaluation provides a basis for extending the investigation to additional enzyme targets, but does not define their inhibitory profiles against AChE, BChE, or HIV-1 RT. Their previously reported cytotoxicity also makes careful definition of enzyme activity and subsequent assessment of biological selectivity essential [8]. Accordingly, the present investigation extends the biological characterisation of these previously reported compounds by examining a defined set of structurally related homologues across three enzyme systems.

A comparative experimental and computational design can help connect these inhibitory profiles to plausible molecular interactions. AChE activity prediction also depends on data preprocessing and dataset characteristics [9]. Biochemical assays establish the activity of each compound against each enzyme, allowing the consequences of alkyl-chain variation to be examined within and across targets. Docking then provides candidate binding geometries for structural interpretation; assessment of the protocol by crystallographic ligand redocking supports evaluation of its ability to reproduce reference poses [10]. Molecular dynamics (MD) adds a time-dependent description of a selected protein–ligand complex, while molecular mechanics/generalised Born surface area (MM/GBSA) calculations provide approximate energetic characterisation within the chosen model. Physicochemical and ADMET predictions complement these analyses by identifying properties relevant to further compound development. Together, these methods allow experimental activity, predicted interactions, and molecular properties to be considered within a common analytical framework.

In this study, the in vitro inhibitory activities of bis-imidazolium derivatives **3e**, **3f**, and **3g** against AChE, BChE, and HIV-1 RT were evaluated and integrated with validated molecular docking and physicochemical and ADMET predictions. Compound **3e** was prioritised on the basis of its experimental inhibition profile, and the HIV-1 RT–**3e** complex was further investigated by MD simulation and MM/GBSA analysis to examine its conformational behaviour and estimated binding energetics. The study aimed to define the inhibitory profile of this homologous series, investigate the relationship between alkyl-chain length and activity, and develop structural hypotheses to guide subsequent experimental assessment and chemical optimisation.

## 2. Results and Discussion

### 2.1. Inhibition of AChE and BChE

**Figure 1:**
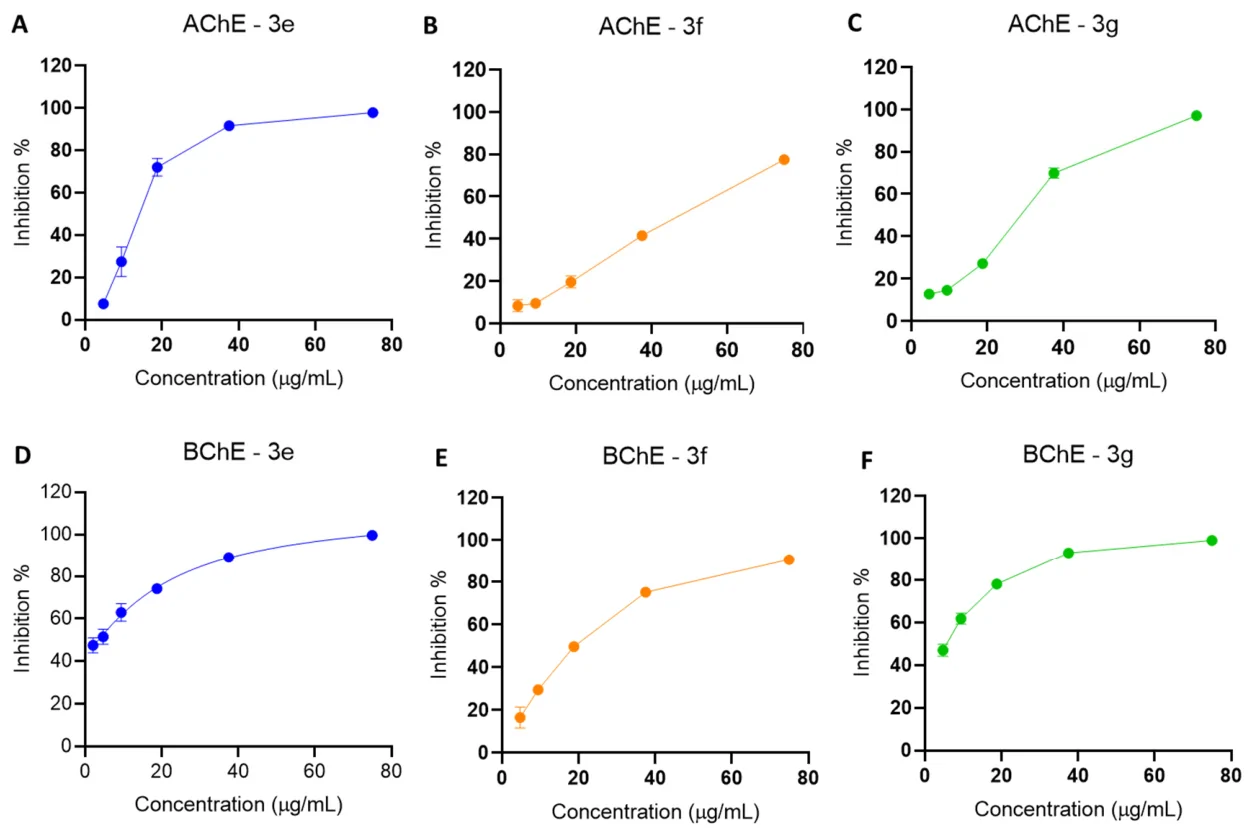
Concentration-dependent inhibition of AChE by (**A**) **3e**, (**B**) **3f**, and (**C**) **3g**, and BChE by (**D**) **3e**, (**E**) **3f**, and (**F**) **3g**. Symbols represent mean inhibition values, and error bars indicate standard deviations of three technical replicates at each concentration within a single experimental run. Connecting lines serve as visual guides. IC_50_ values reported in Table 1 were estimated by nonlinear regression using the “[Inhibitor] vs. normalized response—Variable slope” model in GraphPad Prism version 8.0.1.

The bis-imidazolium derivatives **3e**, **3f**, and **3g** exhibited concentration-dependent inhibition of both AChE and BChE (Figure 1). IC_50_ values were estimated by nonlinear regression using the “[Inhibitor] vs. normalized response—Variable slope” model in GraphPad Prism and are summarized in Table 1. Compound **3e** displayed the lowest IC_50_ against both enzymes, followed by **3g** and **3f**, indicating a consistent activity ranking across the two assays.

For AChE, the IC_50_ values were 13.36, 41.59, and 25.99 µg/mL for **3e**, **3f**, and **3g**, respectively. On a mass-concentration basis, the IC_50_ of **3e** was approximately 3.1-fold lower than that of **3f** and 1.9-fold lower than that of **3g**. The concentration–response profiles likewise showed greater inhibition by **3e** at intermediate concentrations (Figure 1A–C). Donepezil hydrochloride yielded an IC_50_ of 0.048 µg/mL and remained substantially more potent under the assay conditions. Nevertheless, the activity of the investigated derivatives supports further exploration of the bis-imidazolium scaffold as a source of cholinesterase inhibitors. This finding is consistent with recent evidence that imidazolium compounds carrying two positive charges can inhibit AChE, although differences in molecular structure and assay conditions preclude direct potency comparisons between studies [6].

For BChE, **3e** and **3g** yielded For BChE, **3e** and **3g** yielded similar IC_50_ point estimates of 4.95 and 5.32 µg/mL, respectively, whereas **3f** exhibited an IC_50_ of 17.44 µg/mL (Table 1). The small numerical difference between **3e** and **3g** should not be interpreted as an established difference in potency. Thus, the IC_50_ of **3e** was approximately 3.5-fold lower than that of **3f**. The concentration–response profiles supported the stronger activity of **3e** and **3g**, which approached high levels of inhibition at lower concentrations than **3f** (Figure 1D–F). Each compound also exhibited a lower IC_50_ against BChE than against AChE. The AChE/BChE IC_50_ ratios based on mass concentrations were approximately 2.7, 2.4, and 4.9 for **3e**, **3f**, and **3g**, respectively. These values indicate that lower mass concentrations were required to reach 50% inhibition in the BChE assay under the conditions used; however, they should not be interpreted as direct measures of enzyme selectivity because the two enzymes were evaluated using different assay formats. Although **3g** showed the largest difference between its AChE and BChE IC_50_ values, **3e** had the numerically lowest IC_50_ point estimate in both assays.

The shared activity ranking suggests that structural variation within this series affects recognition by both cholinesterases. The higher activity of **3e** relative to the other derivatives also indicates that increasing alkyl-chain length does not produce a uniform improvement in inhibition. One possible explanation is that chain length influences the spatial arrangement of the charged groups and their accommodation within the binding region. This interpretation is consistent with literature emphasizing linker geometry and the positioning of binding motifs in AChE inhibitor design, but remains a hypothesis for the present compounds [11]. Together, these results identify **3e** as the principal compound for further investigation and provide an experimental basis for interpreting the predicted binding poses.

### 2.2. Inhibition of HIV-1 Reverse Transcriptase

**Figure 2:**
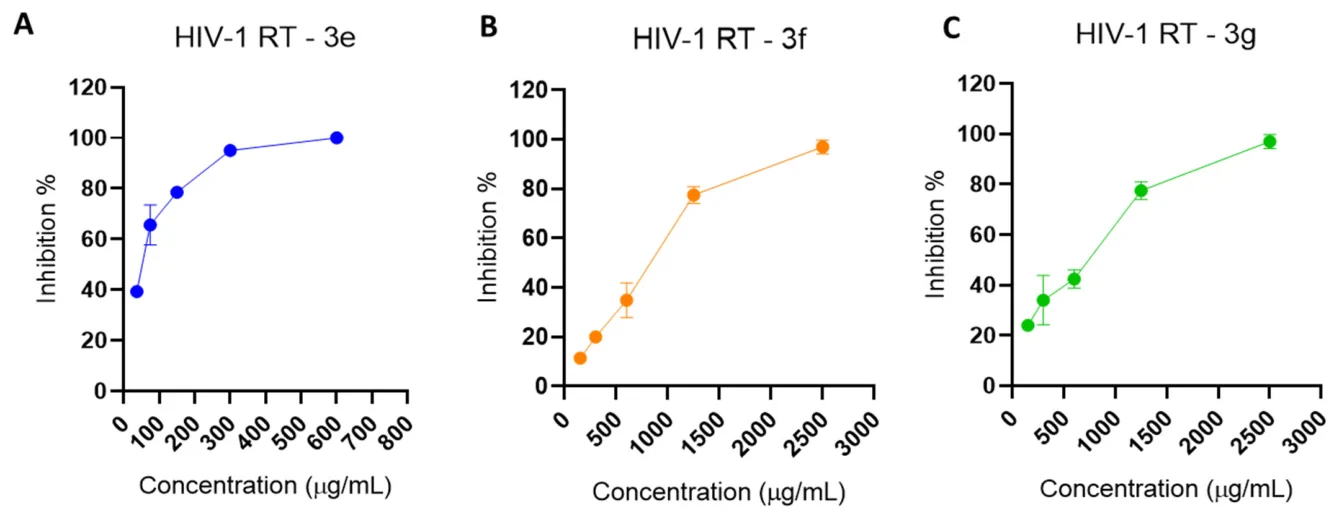
Concentration-dependent inhibition of HIV-1 reverse transcriptase by (**A**) **3e**, (**B**) **3f**, and (**C**) **3g**. Symbols represent mean inhibition values, and error bars indicate standard deviations of three technical replicates at each concentration within a single experimental run. Connecting lines serve as visual guides. IC_50_ values reported in Table 1 were estimated by nonlinear regression using the “[Inhibitor] vs. normalized response—Variable slope” model in GraphPad Prism version 8.0.1.

Compounds **3e**, **3f**, and **3g** exhibited concentration-dependent inhibition of HIV-1 reverse transcriptase (HIV-1 RT) in the non-radioactive ELISA-based assay (Figure 2). IC_50_ values estimated by nonlinear regression using the variable-slope normalized-response model in GraphPad Prism were 50.44, 710.5, and 532.5 µg/mL, respectively (Table 1). Compound **3e** therefore displayed the strongest inhibition within the series, followed by **3g** and **3f**. On a mass-concentration basis, its IC_50_ was approximately 14.1-fold lower than that of **3f** and 10.6-fold lower than that of **3g**.

The concentration–response profiles supported this separation in activity. Compound **3e** approached complete inhibition within a substantially lower concentration range than the other derivatives (Figure 2A), whereas **3f** and **3g** required higher concentrations to achieve comparable responses (Figure 2B,C). Nevirapine, used as the reference inhibitor, produced 95.33% inhibition at 20 µM. This single-concentration reference measurement provides an assay benchmark but does not permit an IC_50_-based potency comparison with the investigated compounds.

The pronounced activity difference between **3e** and the other derivatives indicates that structural variation within the bis-imidazolium series has a substantial influence on RT inhibition. In particular, the stronger activity of **3e** suggests that extending the alkyl chain does not consistently improve inhibitory potency. A possible explanation is that these modifications alter conformational preferences and the positioning of interaction groups within the protein-binding region. Recent structural literature on HIV-1 RT inhibitors emphasizes the importance of ligand geometry, conformational adaptability, and complementary protein–ligand contacts in achieving effective inhibition [12]. These considerations provide a rationale for examining the predicted binding pose of **3e**. However, the IC_50_ values obtained for AChE, BChE, and HIV-1 RT describe inhibitory potency under the assay conditions and do not establish the inhibition mechanism. Docking and MD analyses provide structural hypotheses rather than experimental confirmation of a binding site or inhibition mode. Further enzyme kinetic characterization would be required to distinguish between inhibition mechanisms; therefore, **3e** cannot be classified as an NNRTI on the basis of the present data.

The activity ranking against HIV-1 RT was consistent with that observed for AChE and BChE, identifying **3e** as the most active derivative across all three investigated enzymes. The larger separation between **3e** and the other compounds in the RT assay further supports its prioritization for structural investigation. Accordingly, the HIV-1 RT–**3e** complex was selected for molecular dynamics simulations and MM/GBSA analysis to examine the behavior of its predicted binding pose and the associated interaction-energy components. Collectively, the biochemical findings establish an enzyme-inhibition profile for **3e** that warrants further investigation; antiviral efficacy in infected cells remains to be determined.

The biochemical results should be interpreted in light of the experimental replication scheme. Each enzyme assay was conducted in a single experimental run with three technical replicates per concentration. Accordingly, the reported variability reflects within-run technical variation, and between-experiment reproducibility was not assessed. The IC_50_ values therefore provide preliminary estimates of inhibitory potency under the assay conditions used.

Matched sample-background correction was applied in the BChE assay. However, compound-specific interference was not comprehensively assessed across all three colorimetric assays; therefore, contributions from direct compound absorbance or effects on the detection chemistry cannot be fully excluded.

### 2.3. Molecular Docking Results and Binding-Pose Analysis

The docking protocol was assessed by redocking the co-crystallized reference ligands into their respective binding sites. Nevirapine, donepezil and HUN yielded heavy-atom RMSD values of 0.193, 0.360 and 1.977 Å in HIV-1 RT, AChE and BChE, respectively (Table 2). All three values satisfied the predefined pose-recovery criterion of RMSD < 2.0 Å. The particularly close agreement obtained for nevirapine and donepezil demonstrated effective recovery of their crystallographic orientations, while the HUN pose also remained within the accepted threshold. These results demonstrate reference-pose recovery under the selected docking conditions but do not independently validate the predicted binding modes of compounds **3e**–**3g**. Their docked poses should therefore be regarded as structural hypotheses.

All three compounds yielded negative Vina scores across the investigated targets, with values ranging from −8.586 to −8.237 kcal/mol for HIV-1 RT, −8.588 to −8.413 kcal/mol for AChE, and −8.004 to −7.750 kcal/mol for BChE (Table 2). Compound **3g** produced the most favourable scores for HIV-1 RT and AChE, whereas **3f** ranked first for BChE. Nevertheless, the within-target score ranges were narrow, suggesting that the docking calculations did not strongly discriminate among these closely related derivatives. Nevirapine and donepezil received more favourable scores than the test compounds in their corresponding targets, whereas the three derivatives scored more favourably than HUN in BChE. The discrepancy between docking scores and experimental activity rankings limits affinity-based interpretation of these calculations. Vina scores are approximate scoring-function outputs and do not fully account for solvation, receptor flexibility, or entropy; they should not be interpreted as experimental binding affinities. This integrated approach is consistent with the recognised challenges of translating docking scores into reliable experimental affinity rankings [13].

**Figure 3:**
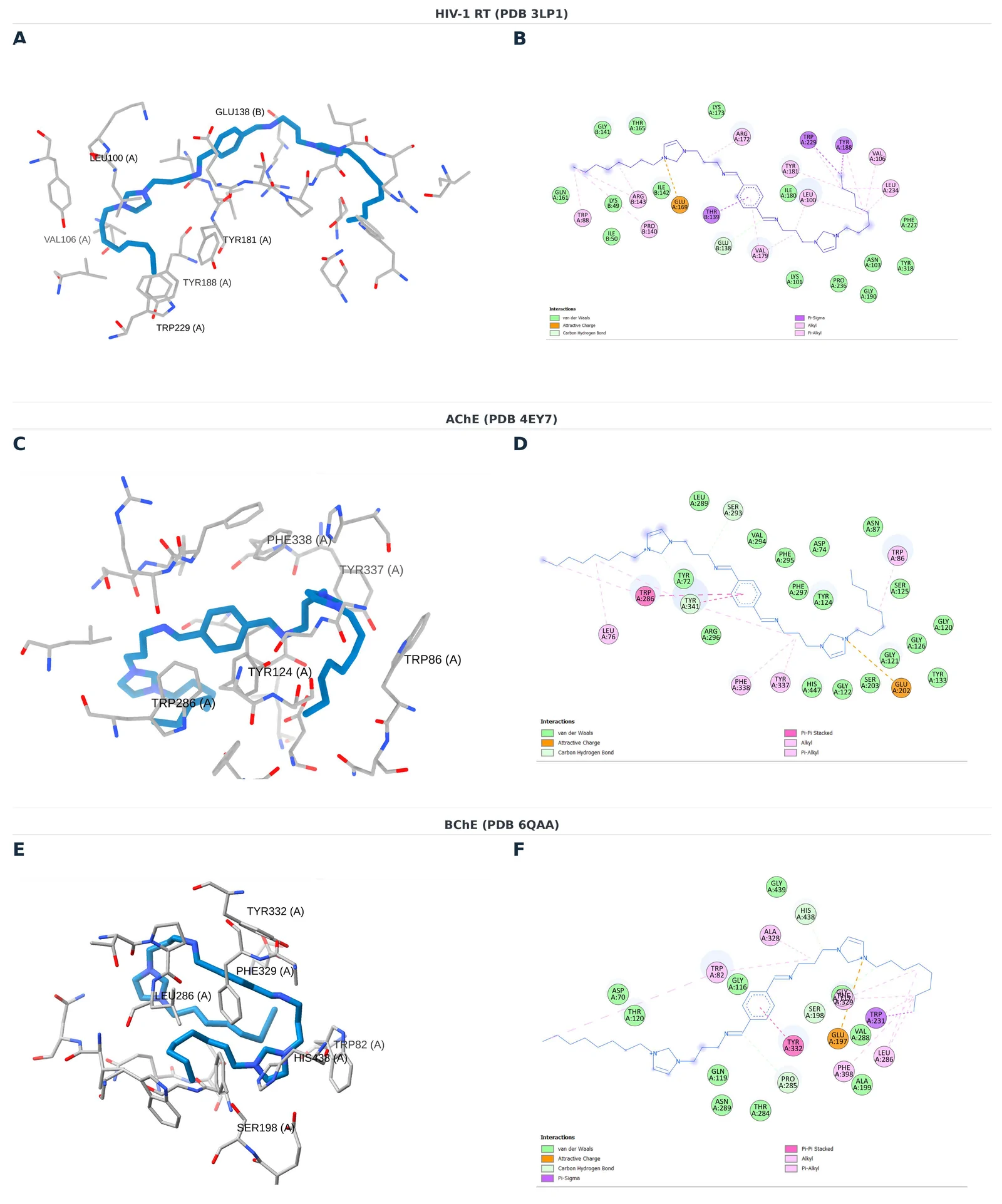
Predicted binding poses and protein–ligand interaction diagrams of compound **3e** in HIV-1 reverse transcriptase (HIV-1 RT; PDB ID: 3LP1), acetylcholinesterase (AChE; PDB ID: 4EY7), and butyrylcholinesterase (BChE; PDB ID: 6QAA). Three-dimensional binding-site views are shown in panels (**A**), (**C**), and (**E**), respectively, and the corresponding two-dimensional interaction diagrams are shown in panels (**B**), (**D**), and (**F**). All panels represent the top-ranked Vina poses. Three-dimensional views were prepared using UCSF ChimeraX v1.10, and two-dimensional interaction diagrams were generated using BIOVIA Discovery Studio Visualizer.

The predicted HIV-1 RT–**3e** pose positioned the ligand within the nevirapine-associated binding region, near Leu100, Val106, Tyr181, Tyr188 and Trp229 of chain A, together with Glu138 of chain B (Figure 3A,B). The interaction diagram assigned π–sigma contacts to Tyr188 and Trp229 of chain A and Thr139 of chain B, accompanied by alkyl, π–alkyl and van der Waals contacts. An attractive-charge interaction with Glu169 of chain A and a carbon–hydrogen-bond contact with Glu138 of chain B provided additional predicted polar contacts. The participation of aromatic and hydrophobic residues in this pose is consistent with the recognition patterns reported for structurally distinct NNRTIs. In a recent study of cyclopentanepyridinone derivatives, docking similarly implicated Tyr181, Tyr188 and Trp229 in ligand recognition within the NNRTI-binding pocket [14]. Thus, the present pose offers a plausible structural explanation for the measured RT inhibition by **3e** and identifies contacts for subsequent trajectory-based evaluation.

In the AChE complex, **3e** occupied the binding gorge near Trp86, Tyr124, Trp286, Tyr337 and Phe338 (Figure 3C,D). The predicted π–π stacking interaction with Trp286 provided an aromatic contact in the peripheral region, while the attractive-charge interaction with Glu202 complemented the ligand’s placement toward the lower gorge. Carbon–hydrogen-bond contacts involving Tyr72, Gly121 and Ser293 further contributed to the predicted interaction network. The distribution of these contacts suggests that the extended scaffold of **3e** can engage more than one region of the gorge. This interpretation accords with a previously described medicinal chemistry strategy, in which suitable linkers connect binding elements capable of occupying catalytic, mid-gorge and peripheral regions of AChE [11]. The predicted arrangement therefore provides a structural hypothesis for the observed AChE inhibition, with aromatic recognition and charge complementarity contributing to the proposed binding mode.

The BChE–**3e** pose showed a related combination of aromatic and polar contacts within a region bordered by Trp82, Ser198, Leu286, Phe329, Tyr332 and His438 (Figure 3E,F). The two-dimensional diagram assigned π–π stacking to Tyr332, a π–sigma interaction to Trp231 and an attractive-charge interaction to Glu197, alongside additional hydrophobic and carbon–hydrogen-bond contacts. Comparable aromatic recognition involving Trp82, Trp231 and Tyr332 has been reported for experimentally active quinazoline BChE inhibitors [15]. The recurrence of these residues across different inhibitor scaffolds supports their relevance to the proposed accommodation of **3e** within the BChE binding region.

Collectively, the docking results suggest that **3e** can adopt target-dependent poses supported by complementary aromatic, hydrophobic and polar contacts. Its predicted recognition within the RT inhibitor-binding region and the cholinesterase gorges provides a coherent structural framework for discussing the experimental inhibition profile. Although **3g** or **3f** received marginally more favourable docking scores, **3e** exhibited the lowest experimental IC_50_ values across the investigated assays. Its prioritisation was therefore guided by the measured activity, with docking supplying a binding hypothesis for further investigation. The HIV-1 RT–**3e** complex was consequently selected for MD and MM/GBSA analyses to examine the persistence of the proposed contacts and the energetic contributions associated with the simulated complex.

### 2.4. Molecular Dynamics Simulation and Dynamic Stability of the HIV-1 RT–**3e** Complex

To determine whether the docking-predicted binding mode of **3e** could be maintained under an explicitly solvated and dynamically flexible environment, the HIV-1 RT–**3e** complex was subjected to a 100 ns molecular dynamics simulation. This analysis was particularly important because HIV-1 reverse transcriptase is a conformationally dynamic p66/p51 heterodimer whose catalytic function depends on coordinated motions involving the fingers, palm, thumb, connection, and RNase H regions. Structural studies have further established that ligand binding to non-catalytic RT sites can influence conformational behavior beyond the immediate binding pocket, thereby contributing to allosteric inhibition [12,16].

**Figure 4:**
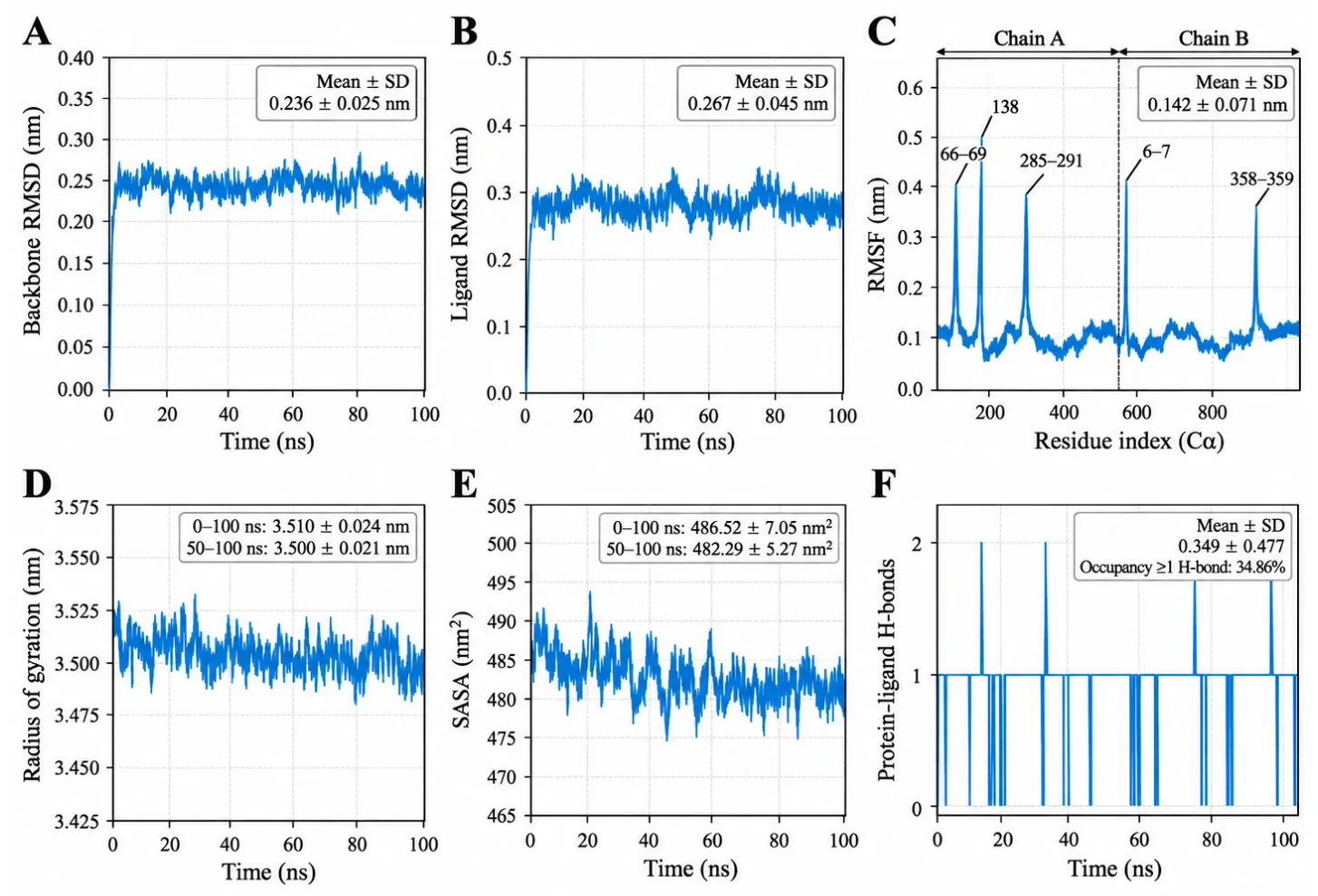
Molecular dynamics analysis of the HIV-1 RT–**3e** complex. (**A**) Protein backbone RMSD, (**B**) ligand RMSD of **3e**, (**C**) Cα RMSF profile, (**D**) radius of gyration, (**E**) solvent-accessible surface area (SASA), and (**F**) protein–ligand hydrogen-bond evolution during the 100 ns simulation. Mean ± SD values are indicated within the corresponding panels; major flexible regions in chains A and B are annotated in panel (**C**).

The backbone RMSD of the RT–**3e** complex remained low throughout the trajectory, with an average value of 0.236 ± 0.025 nm over the complete 100 ns simulation and 0.241 ± 0.022 nm during the final 50 ns (Figure 4A). The close agreement between the full-trajectory and late-stage averages indicates that the complex did not undergo progressive structural drift after equilibration. Independent analysis of the two RT subunits yielded similarly low backbone RMSD values of 0.234 ± 0.036 nm for chain A and 0.204 ± 0.015 nm for chain B, further supporting preservation of the heterodimeric architecture. These observations are consistent with previous structural and simulation-based descriptions of inhibitor-bound HIV-1 RT, in which local and domain-level motions may persist without compromising the overall integrity of the enzyme [16,17].

The ligand also exhibited stable dynamic behavior within its binding environment. Following alignment of the trajectory to the protein backbone, **3e** displayed an average RMSD of 0.267 ± 0.045 nm over the full simulation and 0.262 ± 0.047 nm during the final 50 ns, with a maximum deviation of 0.447 nm (Figure 4B). Importantly, no sustained increase in ligand RMSD was observed toward the end of the trajectory. Instead, **3e** fluctuated within a relatively narrow conformational range, suggesting that the compound remained associated with the docking-defined binding region throughout the simulation. This result strengthens the docking analysis by showing that the predicted binding mode was not restricted to a static minimized structure, but remained compatible with the conformational flexibility of HIV-1 RT.

Residue-level flexibility was further evaluated using Cα RMSF analysis (Figure 4C). The mean RMSF across the protein was 0.142 ± 0.071 nm, indicating generally limited backbone fluctuations. Increased flexibility was localized to discrete segments rather than distributed across the entire structure. In chain A, the most mobile regions were centered around residues 66–69, 135–140, and 285–291, whereas chain B showed higher mobility near residues 6–7 and 358–359. The most flexible individual positions included Glu138, Asn137, Thr139, and Asn136 in chain A, together with Glu6 and Thr7 in chain B. This localized pattern is compatible with the known conformational plasticity of HIV-1 RT. Experimental hydrogen–deuterium exchange studies have shown that NNRTI binding can alter the dynamics of regions located well beyond the canonical inhibitor-binding pocket, including the thumb, connection, RNase H, and p51-associated regions [18]. Similarly, multicopy MD simulations have demonstrated that NNRTI binding can modulate collective motions involving the p66 fingers and thumb subdomains, supporting the concept of long-range allosteric coupling in RT [17].

Global structural compactness was also well maintained. The radius of gyration averaged 3.510 ± 0.024 nm over 100 ns and decreased slightly to 3.500 ± 0.021 nm during the final 50 ns (Figure 4D). A comparable tendency was observed for solvent-accessible surface area, which decreased from an overall average of 486.52 ± 7.05 nm^2^ to 482.29 ± 5.27 nm^2^ during the latter half of the simulation (Figure 4E). The absence of pronounced increases in either radius of gyration (Rg) or solvent-accessible surface area (SASA) argues against progressive structural expansion or unfolding. Instead, the modest decreases observed during the second half of the trajectory are consistent with a limited conformational consolidation of the complex while preserving the global RT architecture.

Protein–ligand hydrogen bonding was comparatively intermittent (Figure 4F). The mean number of direct hydrogen bonds between HIV-1 RT and **3e** was 0.349 ± 0.477, decreasing modestly to 0.257 ± 0.438 during the final 50 ns. At least one hydrogen bond was present in approximately 34.9% of the trajectory, while two simultaneous hydrogen bonds were only rarely observed. Thus, the dynamic stability of **3e** does not appear to depend on a continuously maintained hydrogen-bond network. This is compatible with the broader structural characteristics of HIV-1 RT inhibitor binding, where hydrophobic packing, dispersion interactions, electrostatic complementarity, and shape adaptation can contribute substantially to ligand stabilization in addition to direct polar contacts [12,16].

Within the single 100 ns trajectory, the RMSD, RMSF, Rg, and SASA profiles were consistent with retention of **3e** in the predicted binding region without major structural disruption. However, these observations do not establish convergence, long-term binding stability, or reproducibility across independent simulations. The reported means and standard deviations describe fluctuations within this trajectory, and alternative conformational behavior may not have been sampled. The following MM/GBSA analysis examines the energetic contributions within this sampled configuration.

### 2.5. MM/GBSA Analysis of the HIV-1 RT–**3e** Complex

To complement the structural stability observed during the 100 ns MD simulation, the energetic favorability of the HIV-1 RT–**3e** complex was evaluated using the MM/GBSA approach implemented in gmx_MMPBSA [19,20]. The analysis was performed over the equilibrated 50–100 ns portion of the trajectory using 501 snapshots sampled at 100 ps intervals. MM/GBSA is a widely used end-state approach that combines molecular-mechanical interaction energies with implicit-solvent contributions and provides a computationally efficient framework for characterizing protein–ligand binding energetics, particularly for large biomolecular systems [19,20]. 

The HIV-1 RT–**3e** complex exhibited an overall MM/GBSA binding-energy estimate of −51.04 ± 8.39 kcal/mol (Table 3), indicating an energetically favorable association during the dynamically stable portion of the trajectory. The 501 snapshots were sampled from a single trajectory and are not independent simulation replicates. The reported SD describes variation among sampled frames; temporal correlation may reduce the effective sample size, so the frame-based SEM should not be interpreted as uncertainty across independent simulations or as evidence of convergence. Importantly, this energetic result agrees with the preceding MD analysis, in which both the protein backbone and **3e** maintained low RMSD values during the latter half of the simulation. Thus, the MM/GBSA analysis provides an energetic complement to the structural evidence supporting retention of the docking-derived binding mode.

Inspection of the individual energy components showed that van der Waals interactions constituted the strongest favorable contribution, with Δ*E*_vdW_ of −68.06 ± 10.00 kcal/mol. The nonpolar solvation contribution was also favorable (Δ*G*_SA_ = −8.20 ± 1.44 kcal/mol), indicating that burial of nonpolar surface area contributed to stabilization of the complex. In contrast, the direct electrostatic term was unfavorable (Δ*E*_ele_ = +89.57 ± 19.26 kcal/mol), although this energetic penalty was substantially offset by the favorable generalized Born polar-solvation contribution (Δ*G*_GB_ = −64.35 ± 19.43 kcal/mol).

When grouped according to their physicochemical character, the combined van der Waals and nonpolar contribution amounted to approximately −76.26 kcal/mol, whereas the combined electrostatic and polar-solvation contribution remained net unfavorable at approximately +25.22 kcal/mol. The favorable overall binding-energy estimate therefore appears to arise predominantly from dispersion, close-contact packing, and nonpolar interactions rather than from electrostatic stabilization alone. This energetic profile is consistent with the structural organization of the HIV-1 RT non-nucleoside inhibitor-binding region, which contains a pronounced hydrophobic and aromatic environment formed by residues such as Leu100, Val106, Val179, Tyr181, Tyr188, Phe227, Trp229, and Tyr232 [12,16]. Recent structural reviews continue to emphasize the importance of shape complementarity, aromatic contacts, hydrophobic packing, and conformational adaptability in successful HIV-1 RT allosteric inhibitor recognition [12,16].

The energetic profile also agrees closely with the hydrogen-bond analysis. Although at least one direct protein–**3e** hydrogen bond was present during only approximately 34.9% of the trajectory, **3e** maintained a low ligand RMSD and remained associated with its binding environment throughout the 100 ns simulation. The MM/GBSA data provide an energetic interpretation of this interaction pattern: continuous hydrogen bonding is not required for stable ligand retention when substantial stabilization is supplied by extensive van der Waals and nonpolar contacts. Accordingly, the docking, MD, hydrogen-bond, and MM/GBSA analyses converge on a common interaction model in which **3e** is retained by a distributed network of complementary noncovalent contacts rather than by dependence on a single persistent polar interaction.

**Figure 5:**
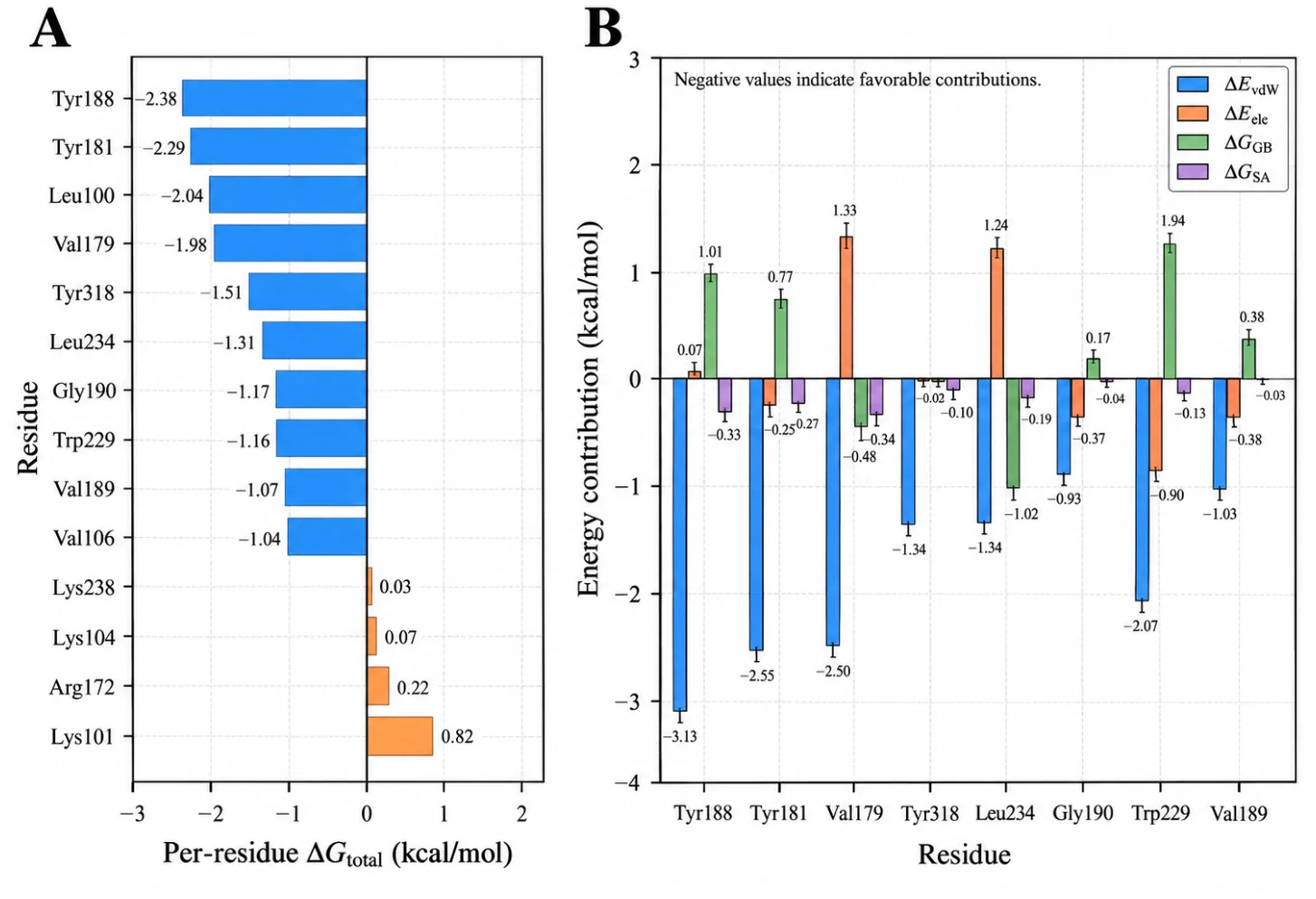
Per-residue MM/GBSA decomposition analysis of the HIV-1 RT–**3e** complex. (**A**) Total per-residue binding-energy contributions of the most favorable and unfavorable residues. (**B**) Energy-component decomposition of the major contributing residues, showing van der Waals, electrostatic, polar solvation, and nonpolar solvation terms. Negative values indicate favorable contributions. Error bars represent the standard deviation (SD).

Per-residue decomposition provided further support for this interpretation (Figure 5). The strongest favorable contributions originated from Tyr188 (−2.38 kcal/mol), Tyr181 (−2.29 kcal/mol), Leu100 (−2.04 kcal/mol), Val179 (−1.98 kcal/mol), Tyr318 (−1.51 kcal/mol), and Leu234 (−1.31 kcal/mol). Additional stabilizing contributions were observed for Gly190, Trp229, Val189, and Val106.

Notably, several of these residues—particularly Leu100, Val106, Val179, Tyr181, Tyr188, and Trp229—are established constituents of the hydrophobic NNRTI-binding environment of HIV-1 RT [12,16]. Their appearance among the most favorable contributors therefore provides an important link between the dynamic energy decomposition and the structural interpretation of the docking pose. Tyr181 and Tyr188 are especially significant because these aromatic residues are central determinants of NNRTI recognition and are also well-known resistance-associated positions whose substitution can markedly alter inhibitor binding [12,21].

The residue-level energy components further showed that the favorable contributions of Tyr188, Tyr181, Val179, Trp229, and Leu234 were dominated largely by negative van der Waals terms, reinforcing the global MM/GBSA result that dispersion and hydrophobic packing represent major stabilizing forces in the RT–**3e** complex. For example, Tyr188 exhibited a van der Waals contribution of approximately −3.13 kcal/mol and a total contribution of −2.38 kcal/mol, while Tyr181 contributed approximately −2.55 kcal/mol through van der Waals interactions and −2.29 kcal/mol overall. Likewise, Trp229 showed a favorable van der Waals term of approximately −2.07 kcal/mol, despite partial compensation by polar-solvation effects.

In contrast, a small number of charged residues displayed mildly unfavorable net contributions. Lys101 contributed approximately +0.82 kcal/mol, while Arg172 contributed approximately +0.22 kcal/mol. In both cases, large direct electrostatic terms were substantially counterbalanced by polar-solvation contributions, illustrating the strong electrostatic–desolvation compensation characteristic of charged residues in continuum-solvent energy decomposition [22,23]. These residues should therefore not be interpreted simply as destabilizing contacts; rather, their net contribution reflects the balance between favorable or unfavorable direct electrostatics and the energetic cost or benefit of solvent reorganization.

The decomposition result is also noteworthy because the strongest stabilizing residues cluster within or adjacent to the established inhibitor-binding environment rather than being randomly distributed across the RT structure. This spatial and energetic convergence increases confidence that the MM/GBSA decomposition is reporting chemically meaningful contacts rather than isolated numerical fluctuations. In particular, the simultaneous involvement of Tyr181, Tyr188, Val179, Leu100, Val106, and Trp229 is consistent with the aromatic–hydrophobic architecture that characterizes NNRTI recognition and provides a plausible structural basis for the favorable ΔE_vdW_ observed for the complete complex [12,16].

The MM/GBSA result is an approximate end-point energy estimate rather than an absolute binding free energy or evidence of high experimental affinity. Its interpretation is limited by single-trajectory conformational sampling, implicit-solvent approximations, and omission of configurational entropy [22,24,25]. For dicationic **3e**, the substantial compensation between electrostatic and polar-solvation terms also makes the estimate sensitive to charge assignment and dielectric treatment. Accordingly, the analysis supports a qualitative description of energetic contributions within the sampled binding pose.

Taken together, the MM/GBSA and per-residue decomposition analyses provide a coherent energetic explanation for the stability observed during MD simulation. Binding of **3e** is dominated by favorable van der Waals and nonpolar contributions, with key stabilization provided by aromatic and hydrophobic residues within the recognized HIV-1 RT inhibitor-binding environment. These findings are consistent with the docking-derived interaction pattern, the low ligand RMSD, and the persistence of the complex despite intermittent hydrogen bonding, collectively supporting a binding model in which **3e** is stabilized primarily through extensive hydrophobic, aromatic, and dispersion-driven contacts within HIV-1 RT.

### 2.6. Predicted Physicochemical and ADMET Properties

The predicted profiles of **3e**–**3g** support the comparative prioritisation of **3e** for further optimisation (Table 4). The SwissADME [26] and Deep-PK [27] outputs showed systematic physicochemical changes with alkyl-chain extension. Compound **3e** combined the lowest molecular weight and flexibility with the lowest consensus logP, while hydrogen-bond counts and TPSA remained unchanged. Both solubility models ranked **3e** as more soluble than its longer-chain analogues, although their absolute estimates differed (Tables S1 and S2). These trends favour **3e** as a starting structure for balancing activity and physicochemical properties, consistent with recent medicinal chemistry strategies that jointly consider lipophilicity, aqueous solubility and membrane permeability [28].

Several screening outcomes were favourable. No PAINS alerts were detected, and Deep-PK returned negative liver-injury classifications for all three compounds. Compound **3e** also received a negative Ames classification, whereas **3f** and **3g** were classified as positive; these Ames predictions had low confidence and therefore represent a preliminary distinction. Using Lipinski’s criteria [29], SwissADME reported one violation for **3e** and **3f**, compared with two for **3g**. Considered alongside the experimental inhibition results, the lower size and lipophilicity burden of **3e** provides a coherent basis for its selection for further investigation. Recent analysis of successful optimisation campaigns likewise highlights the importance of interpreting molecular properties within the relevant target and development context [23].

The predicted low gastrointestinal absorption raises concerns regarding oral exposure, while the lack of predicted BBB permeability limits the rationale for direct central cholinesterase inhibition in Alzheimer’s disease. Cellular exposure relevant to HIV-1 RT inhibition also remains unverified. The high-confidence positive hERG predictions represent a potential cardiac safety concern requiring experimental assessment, rather than an established toxicological outcome. The disagreement between SwissADME and Deep-PK regarding CYP2D6 inhibition further highlights predictive uncertainty (Table 4).

Model applicability also requires caution because these compounds are permanently charged dications with relatively high molecular weights and numerous rotatable bonds. Their representation within the models’ training domains has not been established, and server-reported confidence does not independently demonstrate applicability to this chemical series. Accordingly, these predictions identify potential development liabilities rather than establish pharmacokinetic or safety profiles. Additional outputs, confidence categories, and training-range flags are provided in Tables S1 and S2.

## 3. Materials and Methods

### 3.1. Compounds and Chemical Structures

Three bis-imidazolium bromide derivatives, designated **3e**, **3f**, and **3g**, were evaluated in this study. The compounds had previously been synthesised and characterised by our research group, as reported previously [8]. Samples from that original study were used for the present enzyme inhibition experiments. Their chemical identities and structural characterisation were based on the Fourier-transform infrared and proton and carbon-13 nuclear magnetic resonance spectroscopy data reported in the original publication [8].

**Figure 6:**
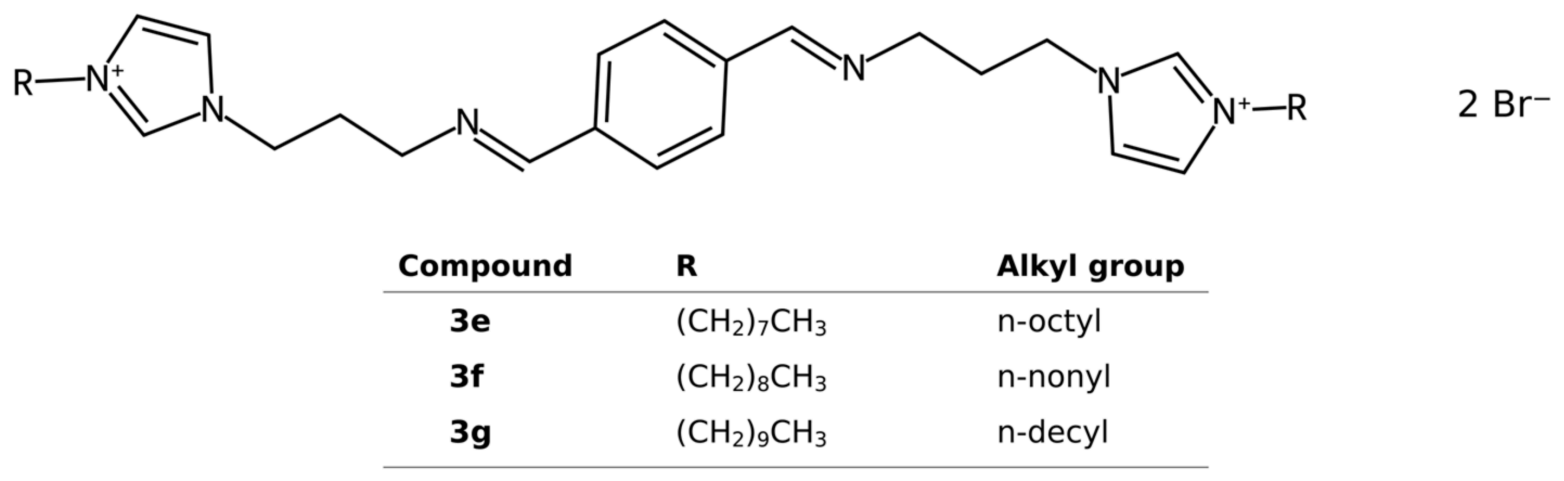
Chemical structures of bis-imidazolium bromide derivatives **3e**–**3g**. The common scaffold is shown with R representing n-octyl (**3e**), n-nonyl (**3f**), or n-decyl (**3g**) groups. Structures were redrawn from the original publication [8].

The compounds share a common bis-imidazolium framework and differ in their N-alkyl substituents, with octyl, nonyl, and decyl groups in **3e**, **3f**, and **3g**, respectively (Figure 6). The original compound designations were retained throughout the study. Stock solutions of the test compounds were prepared in 10% dimethyl sulfoxide (DMSO; Merck, Darmstadt, Germany; catalogue no. D8418).

### 3.2. Acetylcholinesterase Inhibition Assay

AChE inhibitory activity was determined using a modified Ellman colorimetric assay [30]. Recombinant human AChE (Sigma-Aldrich, St. Louis, MO, USA; catalogue no. C1682) was used as the enzyme source, and donepezil hydrochloride (Sigma-Aldrich; ≥98% purity by HPLC; CAS no. 120011-70-3) served as the positive control.

The assay was performed in 96-well microplates. Each well contained 50 μL of Tris-HCl buffer (50 mM, pH 8.0), 125 μL of 5,5′-dithiobis(2-nitrobenzoic acid) solution (DTNB; 3 mM; Merck, catalogue no. D8130), 25 μL of AChE solution (0.2 U/mL), and 25 μL of test compound solution at the indicated concentration. The mixture was preincubated at room temperature for 15 min. Subsequently, 25 μL of acetylthiocholine iodide solution (15 mM) was added to initiate the reaction, yielding a total reaction volume of 250 μL. Following a further 15 min incubation at room temperature, absorbance was measured at 405 nm using a Multiskan SkyHigh microplate spectrophotometer (Thermo Scientific, Singapore). The final DMSO concentration was maintained at 0.4% (*v*/*v*) in the 250 µL reaction volume at all tested compound concentrations. Vehicle controls and corresponding enzyme-free sample blanks contained the same final DMSO concentration.

Percentage inhibition was calculated using the following equation:

Inhibition (%) = [(A_control_ − A_sample_)/A_control_] × 100

where A_control_ and A_sample_ represent the absorbance values of the negative control and test compound wells, respectively. Concentration–response analysis and IC_50_ determination are described in Section 2.5.

### 3.3. Butyrylcholinesterase Inhibition Assay

BChE (EC 3.1.1.8) inhibitory activity was evaluated using the Butyrylcholinesterase Activity Kit (Colorimetric; Sigma-Aldrich, catalogue no. MAK406), following the manufacturer’s protocol with an adaptation for testing small-molecule inhibitors. The kit-supplied BChE preparation derived from equine serum (Sigma-Aldrich, component MAK406C) was used as the enzyme source. It was reconstituted in 20 µL of BChE Assay Buffer and diluted 50-fold before use. The manufacturer did not specify activity per vial; therefore, the enzyme loading is reported by preparation and dilution rather than in U/mL. Test compounds were added to the assay wells at the indicated concentrations. Each test well received 10 μL of the diluted BChE solution, and the volume was adjusted to 95 μL with BChE Assay Buffer. Vehicle-control wells contained the same volume of vehicle and diluted BChE but no test compound. The final DMSO concentration, including the contribution from the substrate solution, was maintained at 0.4% (*v*/*v*) in the 200 µL reaction volume at all tested compound concentrations. Vehicle controls and corresponding enzyme-free sample blanks contained the same final DMSO concentration. Parallel sample-background control wells were prepared for background correction.

DTNB solution (5 μL) was added to the test, vehicle-control, and corresponding sample-background control wells. The plate was incubated for 10 min at room temperature, protected from light. The kit substrate was diluted 120-fold with BChE Assay Buffer, briefly vortexed, and kept on ice. The reaction was initiated by adding 100 μL of diluted substrate to the test and vehicle-control wells. The corresponding sample-background control wells received 100 μL of BChE Assay Buffer instead of substrate, giving a final volume of 200 μL per well. After shaking the plate for 10 s, absorbance was measured immediately at 412 nm in kinetic mode for 20–30 min at room temperature.

A TNB standard curve (0–30 nmol per well) was prepared according to the manufacturer’s instructions. After subtraction of the zero-standard reading, two time points within the linear range were selected. TNB formation was determined from the standard curve, and the matched sample-background value was subtracted from each test and vehicle-control value. Percentage inhibition was calculated from the corrected BChE activity relative to the vehicle control. IC_50_ determination is described in Section 2.5.

### 3.4. HIV-1 Reverse Transcriptase Inhibition Assay

The inhibitory activities of compounds **3e**, **3f**, and **3g** against HIV-1 RT were evaluated using the colorimetric Reverse Transcriptase Assay (Roche Diagnostics; Mannheim, Germany; Version 16.0; catalogue no. 11 468 120 910), according to the manufacturer’s inhibitor-determination procedure. Nevirapine (Sigma-Aldrich, catalogue no. SML0097) was used as the reference inhibitor. Test compounds and nevirapine were prepared in DMSO at the indicated concentrations.

The assay quantifies RT activity through the synthesis of DNA using the kit-supplied poly(A)·oligo(dT)_15_ template–primer hybrid and the incorporation of biotin- and digoxigenin-labelled nucleotides. The resulting labelled DNA is captured by streptavidin-coated microplate modules and detected using peroxidase-conjugated anti-digoxigenin antibody (anti-DIG-POD) and ABTS substrate. The colorimetric signal is proportional to RT activity. Earlier colorimetric RT assays also used biotin- and digoxigenin-labelled nucleotide incorporation [31].

For each reaction, 4–6 ng of HIV-1 RT in 20 μL of lysis buffer was added to a reaction tube, followed by 20 μL of test-compound or nevirapine solution and 20 μL of reaction mixture. The resulting 60 μL reaction was incubated at 37 °C for 1 h. The final DMSO concentration was maintained at 0.4% (*v*/*v*) in the 60 µL RT reaction at all tested compound concentrations. Vehicle controls and corresponding enzyme-free sample blanks contained the same final DMSO concentration. An RT-free reaction, prepared using lysis buffer in place of the enzyme, was included as the background blank. An RT-containing reaction with vehicle in place of inhibitor served as the inhibitor-free activity control.

After incubation, 60 μL of each reaction was transferred to the streptavidin-coated microplate modules and incubated at 37 °C for 1 h. The liquid was removed, and the wells were washed five times with 250 μL of washing buffer (Solution 6). Anti-DIG-POD working solution (200 μL; 200 mU/mL, Solution 5a) was added to each well, followed by incubation at 37 °C for 1 h. The wells were then washed five times with 250 μL of washing buffer, allowing 30 s for each washing step. ABTS substrate solution (200 μL) was added to each well, and color was allowed to develop at 15–25 °C for 10–30 min. Absorbance was measured at 405 nm with a reference wavelength of 490 nm. Inhibition was calculated relative to the inhibitor-free RT control after correction for the RT-free background; IC_50_ determination is described in Section 2.5.

### 3.5. IC_50_ Determination and Data Analysis

For each enzyme assay, each concentration was tested in three technical replicates within a single experimental run. No independent experimental runs were performed. Percentage inhibition data are presented as the mean ± standard deviation (SD) of the three technical replicates; these SD values describe within-run technical variability rather than between-experiment reproducibility. For AChE, percentage inhibition was calculated from the absorbance of the compound-treated wells relative to the vehicle control, as described in Section 2.2. For BChE, TNB formation was quantified using the kit standard curve, corrected against the corresponding sample-background control, and compared with the vehicle control. For HIV-1 RT, the signal was corrected against the RT-free background and normalized to the inhibitor-free RT control.

Concentration–response plots were generated for each compound. IC_50_ values were estimated by nonlinear regression using the “[Inhibitor] vs. normalized response—Variable slope” model in GraphPad Prism version 8.0.1. The 95% confidence intervals for IC_50_ estimates were calculated using the profile likelihood method. These intervals describe uncertainty in the fitted estimates within the single experimental run and do not quantify between-experiment reproducibility. IC_50_ was defined as the concentration corresponding to 50% inhibition. All IC_50_ values are reported in µg/mL, consistent with the concentration units used in Table 1. Nevirapine was tested as a reference inhibitor at 20 µM; an IC_50_ value was not determined for this control.

### 3.6. Protein and Ligand Preparation

The crystal structures of acetylcholinesterase (AChE; PDB ID: 4EY7, chain A), butyrylcholinesterase (BChE; PDB ID: 6QAA, chain A), and HIV-1 reverse transcriptase (HIV-1 RT; PDB ID: 3LP1, chains A and B) were retrieved from the Protein Data Bank [32]. Water molecules were removed, and the co-crystallized ligands were retained separately for redocking validation. Missing residues and atoms were repaired using PDBFixer. The receptor protonation states were assigned at pH 7.4, and the prepared receptor structures were converted to PDBQT format using Meeko 0.7.1 [33].

The three-dimensional structures of compounds **3e**, **3f**, and **3g** were generated using RDKit with the ETKDGv3 method [34,35] and energy-minimized using the MMFF94s force field [36,37]. Each compound was modeled in its dicationic (+2) state; bromide counterions were omitted. The ligands were converted to PDBQT format using Meeko 0.7.1. The co-crystallized reference ligands donepezil (E20), HUN, and nevirapine (NVP) were prepared using the same ligand-preparation workflow for redocking validation.

### 3.7. Molecular Docking and Redocking Validation

Molecular docking was performed with AutoDock Vina 1.2.7 using the Vina scoring function [10,38]. The prepared receptors were treated as rigid, while ligand torsional degrees of freedom were sampled during docking. Docking calculations used an exhaustiveness of 64, a maximum of 20 poses per run, eight CPU threads, and random seed 20260929.

For AChE, the search box was centered at (−14.108, −43.833, 27.670) Å; for BChE, at (18.578, 42.300, 41.038) Å; and for HIV-1 RT, at (10.175, 13.836, 17.776) Å. Each box measured 40 × 40 × 40 Å. The top-ranked pose was selected for structural comparison. Docking scores are reported in kcal mol^−1^ as Vina ranking scores and were not interpreted as experimentally measured binding free energies.

Redocking validation was performed by redocking each co-crystallized reference ligand into its corresponding receptor binding site. The RMSD between the crystallographic and redocked ligand coordinates was calculated after hydrogen atoms were removed, without independently superimposing the ligand structures, to preserve their receptor-frame alignment. Symmetry-equivalent atom mappings were considered during RMSD calculation. An RMSD of ≤2.0 Å was used as the criterion for acceptable pose reproduction. The redocking RMSD values are reported with the docking results.

For interaction analysis, a protein residue was considered to contact a ligand when at least one pair of ligand–protein heavy atoms was within 4.0 Å. Contacts were compared with those of the corresponding crystallographic reference ligand. Ligand nitrogen–protein oxygen distances were examined as potential polar contacts; distance alone was not considered sufficient to assign a hydrogen bond or a specific interaction mechanism.

Numerical analysis was conducted using NumPy 2.5.3 and SciPy 1.18.1, and data tabulation was performed using pandas 3.0.6.

### 3.8. Molecular Dynamics Simulation of the HIV-1 RT–**3e** Complex

Molecular dynamics (MD) simulation was used to examine the time-dependent stability and interaction behavior of the HIV-1 RT–**3e** complex selected from the experimental and docking results. Compound **3e** showed the strongest HIV-1 RT inhibition among the tested derivatives, with an IC_50_ of 50.44 µg mL^−1^; this experimental result, considered together with the docking analysis, motivated its selection for MD simulation. Only the HIV-1 RT–**3e** complex was subjected to MD simulation, using a single 100 ns trajectory; no independent MD replicate was performed.

The system was prepared and simulated using GROMACS 2024.1 [39,40]. The protein was described with the AMBER99SB-ILDN force field [41], while ligand parameters were generated using ACPYPE [42] with the General AMBER Force Field (GAFF) [43] and AM1-BCC partial charges [44,45]. No dedicated validation of the GAFF/AM1-BCC parameters was performed for dicationic **3e**; this introduces uncertainty into the MD and MM/GBSA results. The complex was placed in a triclinic periodic box with a minimum solute-to-box-boundary distance of 1.0 nm and solvated with explicit TIP3P water [46]. Counterions were added to neutralize the system, and NaCl was added to a final concentration of 0.15 M.

Long-range electrostatic interactions were calculated using the Particle Mesh Ewald (PME) method [47]. Coulombic and van der Waals interactions were treated with a 1.0 nm cutoff. Covalent bonds were constrained using the LINCS algorithm [48], and a 2 fs integration time step was applied. The solvated system was energy-minimized using the steepest-descent algorithm. Equilibration comprised a 125 ps NVT stage at 300 K using the V-rescale thermostat [49], followed by a 125 ps NPT stage at 1 bar using the Parrinello–Rahman barostat [50]. The sequential NVT and NPT stages were intended to allow initial thermal relaxation and adjustment of the solvent density, respectively. These short equilibration stages do not by themselves establish complete equilibration or conformational convergence. A 100 ns production simulation was then performed under periodic boundary conditions. The production phase was run on a CUDA-enabled GPU on the TRUBA high-performance computing system, provided by TUBITAK ULAKBIM, Ankara, Türkiye. Trajectory coordinates were saved at 2 ps intervals.

The trajectory was analyzed for protein-backbone and ligand RMSD, residue-level RMSF, Rg, SASA, and protein–ligand hydrogen-bond behavior. These analyses were used to characterize structural stability, flexibility, compactness, solvent exposure, and the persistence of polar interactions during the simulation.

### 3.9. MM/GBSA Analysis

To estimate the binding-energy components of the HIV-1 RT–**3e** complex, MM/GBSA calculations were performed using gmx_MMPBSA version 1.7.0 [19]. The analysis was carried out over the equilibrated final 50 ns of the 100 ns production trajectory (50–100 ns). A total of 501 snapshots, sampled at 100 ps intervals, were included in the calculation. The receptor and ligand topologies were derived from the same parameterization scheme used during the MD simulations, with AMBER99SB-ILDN parameters for the protein and GAFF parameters for compound **3e**.

The binding-energy estimate was expressed as:

Δ*G*_bind_ = Δ*E*_vdW_ + Δ*E*_ele_ + Δ*G*_GB_ + Δ*G*_SA_

where Δ*E*_vdW_ and Δ*E*_ele_ denote van der Waals and electrostatic contributions, respectively, and Δ*G*_GB_ and Δ*G*_SA_ represent polar and nonpolar solvation contributions. The polar solvation term was calculated using the Generalized Born model with igb = 5, and the salt concentration was set to 0.150 M. Default dielectric settings of gmx_MMPBSA/AmberTools were retained for the GB calculation. The nonpolar solvation contribution was estimated from the solvent-accessible surface area term.

Per-residue energy decomposition was additionally performed using idecomp = 2 to identify amino-acid residues contributing favorably or unfavorably to ligand binding. Residues located within 6 Å of the ligand were included in the decomposition analysis. Energy components were summarized as mean ± standard deviation across the sampled snapshots.

Configurational entropy was not explicitly calculated. Accordingly, the reported MM/GBSA values were interpreted as end-point binding-energy estimates and mechanistic energetic descriptors, rather than as rigorous absolute experimental binding free energies [22,51].

### 3.10. Physicochemical, Drug-Likeness, and ADMET Predictions

The physicochemical properties and ADME profiles of compounds **3e**–**3g** were calculated using SwissADME [26], and complementary pharmacokinetic and toxicity predictions were obtained using Deep-PK [27]. Molecular graphs were taken from the first SDF record of each compound in the docking dataset and converted to SMILES. Bromide counterions were excluded, while the +2 formal charge and encoded imine stereochemistry were retained. Molecular weight, hydrogen-bond counts, topological polar surface area and rotatable-bond counts were independently checked using RDKit 2026.03.6. Source-specific descriptor values were retained. Deep-PK classification outputs were recorded with their displayed confidence categories, numerical scores and endpoint-specific MW training-range flags. Predictions with implausible numerical values or inconsistent unit/scale labels were preserved in the complete output record and excluded from quantitative interpretation. Analyses were treated as computational screening results rather than experimental pharmacokinetic or safety measurements.

## 4. Conclusions

In this study, bis-imidazolium derivatives **3e**–**3g** showed concentration-dependent inhibition of AChE, BChE, and HIV-1 RT, with **3e** displaying the lowest IC_50_ values within the tested series (13.36, 4.95, and 50.44 µg/mL, respectively). Redocking reproduced the reference-ligand poses, while docking scores showed limited ability to distinguish the closely related compounds and did not fully match the experimental activity ranking. In the single 100 ns MD trajectory, **3e** remained within the predicted HIV-1 RT binding region; the MM/GBSA estimate was −51.04 ± 8.39 kcal/mol, without an explicit configurational-entropy contribution. ADMET predictions indicated low gastrointestinal absorption, no predicted blood–brain barrier permeation, and a potential hERG-blockade liability. Taken together, the results identify **3e** as the most active derivative in this series and provide a basis for further evaluation. These findings do not establish cellular antiviral efficacy, experimental binding affinity, or reproducibility across independent MD trajectories. Cellular antiviral activity and selectivity remain unconfirmed and require cell-based antiviral testing with matched cytotoxicity assessment.

## Figures and Tables

**Table 1:** In vitro inhibitory activities of compounds **3e**, **3f**, and **3g** against AChE, BChE, and HIV-1 reverse transcriptase.

Compound	AChE IC_50_ (95% CI)	BChE I C_50_ (95% CI)	HIV-1 RT IC_50_ (95% CI)
**3e**	13.36 (12.44–14.34)	4.950 (3.571–6.248)	50.44 (43.30–57.52)
**3f**	41.59 (36.87–47.09)	17.44 (16.03–18.96)	710.5 (601.4–834.6)
**3g**	25.99 (22.01–30.44)	5.322 (4.587–6.028)	532.5 (394.3–704.3)
Donepezil hydrochloride	0.048	—	—

Values are IC_50_ estimates with profile likelihood 95% confidence intervals in parentheses, expressed in µg/mL. IC_50_, concentration required to inhibit enzyme activity by 50%; AChE, acetylcholinesterase; BChE, butyrylcholinesterase; HIV-1 RT, HIV-1 reverse transcriptase. Donepezil hydrochloride was the reference inhibitor in the AChE assay. Nevirapine produced 95.33% HIV-1 RT inhibition at 20 µM; an IC_50_ value was not available. —, value not available. IC_50_ values are regression-derived point estimates from concentration–response data obtained in a single experimental run with three technical replicates per concentration. They do not represent mean IC_50_ values from independent experiments.

**Table 2:** Top-ranked Vina docking scores of compounds **3e**, **3f**, and **3g** and the corresponding reference ligands, together with reference-ligand redocking RMSD values.

Target	PDB ID	Ligand	Vina Score (kcal/mol)	Redocking RMSD (Å)
HIV-1 RT	3LP1	**3e**	−8.237	—
		**3f**	−8.484	—
		**3g**	−8.586	—
		Nevirapine (NVP)	−11.111	0.193
AChE	4EY7	**3e**	−8.468	—
		**3f**	−8.413	—
		**3g**	−8.588	—
		Donepezil (E20)	−12.187	0.360
BChE	6QAA	**3e**	−7.936	—
		**3f**	−8.004	—
		**3g**	−7.750	—
		HUN	−7.129	1.977

Scores correspond to the top-ranked poses obtained using Vina scoring. Redocking RMSD values were calculated for reference-ligand heavy atoms relative to crystallographic coordinates, without additional ligand superposition. —, not applicable.

**Table 3:** MM/GBSA energy components for the HIV-1 RT–**3e** complex calculated over the final 50 ns of the MD trajectory.

Energy Component	Mean ± SD (kcal/mol)	SEM (kcal/mol)	Contribution
Δ*E*_vdW_	−68.06 ± 10.00	0.45	Favorable
Δ*E*_ele_	+89.57 ± 19.26	0.86	Unfavorable
Δ*G*_GB_	−64.35 ± 19.43	0.87	Favorable
Δ*G*_SA_	−8.20 ± 1.44	0.06	Favorable
Δ*G*_bind_	−51.04 ± 8.39	0.38	Overall favorable

Values were obtained from 501 snapshots collected between 50 and 100 ns. Configurational entropy was not included; therefore, Δ*G*_bind_ should be interpreted as an MM/GBSA end-point binding-energy estimate rather than a rigorous absolute binding free energy.

**Table 4:** Selected physicochemical and ADMET predictions for compounds **3e**–**3g**.

Property or Endpoint	3e	3f	3g
Molecular weight (g/mol) ^a^	574.89	602.94	630.99
H-bond acceptors/donors	2/0	2/0	2/0
TPSA (Å^2^)	42.34	42.34	42.34
Rotatable bonds	24	26	28
Consensus logP	5.21	5.91	6.73
Lipinski violations ^b^	1	1	2
GI absorption	Low	Low	Low
BBB permeant	No	No	No
P-gp substrate	Yes	Yes	Yes
CYP2D6 inhibition (S/D) ^c^	No/Yes (H)	No/Yes (H)	No/Yes (H)
Liver injury I (DILI) ^d^	Negative (M)	Negative (M)	Negative (M)
Ames mutagenicity ^d^	Negative (L)	Positive (L)	Positive (L)
hERG blockade ^d^	Positive (H)	Positive (H)	Positive (H)
PAINS alerts	0	0	0

^a^ Values refer to organic dications (+2), without bromide counterions. ^b^ MW > 500 g/mol for all compounds; **3g** additionally has MLOGP > 4.15. ^c^ S/D, SwissADME/Deep-PK; both predictions are shown. ^d^ Deep-PK; H, M and L denote high, medium and low server confidence. Other entries are from SwissADME. Positive/negative denotes an endpoint-specific predicted class. Additional outputs and model limitations are provided in Tables S1 and S2.

## Data Availability

The data supporting the findings of this study are available in the article and its Supplementary Materials. Additional raw assay data and the molecular dynamics trajectory and analysis files are available from the corresponding author upon reasonable request.

## Supplementary Materials

The following supporting information can be downloaded at: https://www.mdpi.com/article/10.3390/molecules31214006/s1, Table S1: Additional SwissADME descriptors and screening outputs for compounds **3e–3g**; Table S2: Complementary Deep-PK predictions and selected model limitations for compounds **3e–3g**.
