# Supplementary material for: Integrated Biochemical and Computational Evaluation of Bis-Imidazolium Derivatives 3e–3g in Cholinesterase and HIV-1 Reverse Transcriptase Assays
Source: Molecules. 2026 Nov 6;31(21):4006. doi: 10.3390/molecules31214006 (PMC13764758; doi:10.3390/molecules31214006)
Supplement: Supplementary file 1 [file molecules-31-04006-s001.zip › molecules-4656106-supplementary.pdf]

Table S1. Additional SwissADME descriptors and screening outputs for compounds 3e–3g.

**Property or endpoint**	**3e**	**3f**	**3g**
Molecular weight (g/mol)	574.89	602.94	630.99
Formal charge	+2	+2	+2
H-bond acceptors	2	2	2
H-bond donors	0	0	0
TPSA (Å^2)	42.34	42.34	42.34
Rotatable bonds	24	26	28
GI absorption	Low	Low	Low
BBB permeant	No	No	No
P-gp substrate	Yes	Yes	Yes
CYP1A2 inhibitor	No	No	No
CYP2C19 inhibitor	No	No	No
CYP2C9 inhibitor	No	No	No
CYP2D6 inhibitor	No	No	No
CYP3A4 inhibitor	No	No	No
Lipinski violations	1 (MW)	1 (MW)	2 (MW, MLOGP)
Veber violations	1 (rotors)	1 (rotors)	1 (rotors)
Bioavailability score	0.55	0.55	0.17
PAINS alerts	0	0	0
Brenk alerts	2	2	2
Synthetic accessibility	4.99	5.25	5.51
Log Po/w (iLOGP1.5)	-3.01	-2.58	-1.57
Log Po/w (XLOGP3)	8.19	9.28	10.36
Log Po/w (WLOGP)	7.60	8.38	9.16
Log Po/w (SILICOS-IT)	9.51	10.36	11.22
Log S (ESOL)	-7.26	-7.98	-8.69
Log S (Ali)	-8.94	-10.07	-11.19
Log S (SILICOS-IT)	-11.73	-12.51	-13.28
MLOGP	3.77	4.13	4.47

MW refers to organic dications without bromide counterions. The formal charge is +2. Bioavailability scores are model scores, not measured oral bioavailability. Lipophilicity and solubility estimates vary by model; aqueous behaviour of the tested salts requires experimental measurement. Brenk alerts are imine_1 and quaternary_nitrogen_1.

Table S2. Complementary Deep-PK predictions and selected model limitations for compounds 3e–3g.

**Property or endpoint**	**3e**	**3f**	**3g**
logD at pH 7.4	4.04	4.53	5.00
logS (mol/L)	-3.02	-3.28	-3.59
Human intestinal absorption	Absorbed (L)	Non-Absorbed (L)	Non-Absorbed (L)
BBB permeation	Non-Penetrable (H)†	Non-Penetrable (H)†	Non-Penetrable (H)†
P-gp substrate	Yes (H)	Yes (H)	Yes (H)
P-gp inhibition	Yes (H)	Yes (H)	Yes (H)
CYP2D6 inhibition	Yes (H)	Yes (H)	Yes (H)
Ames mutagenicity	Negative (L)	Positive (L)	Positive (L)
hERG blockade	Positive (H)	Positive (H)	Positive (H)
Drug-induced liver injury I	Negative (M)	Negative (M)	Negative (M)
Liver injury II	Negative (L)	Negative (M)	Negative (M)
Micronucleus	Positive (H)†	Positive (H)†	Positive (H)†
Skin sensitisation	Positive (M)†	Positive (M)†	Positive (M)†
OCT2 inhibition	Yes (H)†	Yes (H)†	Yes (H)†
Elimination half-life	<3 h (L)†	≥3 h (L)†	≥3 h (L)†
Clearance output‡	4.28†	4.13†	3.94†

H, M and L denote the high, medium and low confidence categories displayed by Deep-PK. † MW is outside the reported endpoint training range. ‡ Clearance is preserved as a raw output; the result page labels the scale Log (ml/min/kg), whereas the theory page describes ml/min/kg. Clearance and the flagged half-life outputs are not interpreted quantitatively. MW-range checks do not establish a complete applicability domain.
